# Spontaneous Rupture of the Anterior Vaginal Wall during the First Stage of Labour

**DOI:** 10.1155/2012/786753

**Published:** 2012-06-03

**Authors:** Meleesa Joy Schultz, Triveni Nanda

**Affiliations:** ^1^Department of Obstetrics and Gynaecology, Hornsby Ku-ring-gai Hospital, Palmerston Road, Hornsby, NSW 2077, Australia; ^2^Department of Obstetrics and Gynaecology, Canberra Hospital, Yamba Drive, Garran, ACT 2605, Australia

## Abstract

The risk of uterine rupture during attempted trial of labor after caesarean delivery (TOLAC) is well documented. However, vaginal rupture (in the absence of obstructed labour) is exceptionally uncommon. Below is described the rare case of a 37-year-old multiparous woman attempting TOLAC, who suffered a vaginal—rather than uterine—rupture, during the first stage of spontaneous labour. This case is an important reminder to obstetricians that concealed ruptures of both the vagina and uterus do occur and must be considered in clinical situations where another explanation is not apparent.

## 1. Introduction

The risk of uterine rupture during a trial of labor after caesarean delivery (TOLAC) is well documented [1]. Below is described the rare case of a patient attempting TOLAC who suffered a vaginal—rather than uterine—rupture, during the first stage of spontaneous labour.

## 2. Case Report

A healthy 37-year-old multiparous woman, gravida two para one, presented to the birthing unit in spontaneous labour at term. Three years prior, she had an emergency lower-segment caesarean section (LSCS) for failure to progress in labour, at 8 cm cervical dilatation. She had no other history of gynaecological surgery. On presentation, she was contacting every three minutes, with membranes intact and a reassuring cardiotocograph. An amniotomy was performed two hours later, at 8 cm cervical dilatation and station 2, producing copious clear liquor. No abnormalities were noted.

Two minutes after the amniotomy, a ten-minute foetal bradycardia occurred (to 77 bpm), and the patient was transferred to theatres for an emergency LSCS. She was not in significant pain and had only scant vaginal bleeding. Due to the urgency of the situation, neither abdominal nor vaginal examinations were performed at that time. A live female infant was delivered 15 mins later, weighing 3285 g. The baby was dusky, with blood throughout her airways, and cord pHs of 6.97 (arterial) and 7.15 (venous). Despite this, the baby resuscitated well and had Apgars of eight (at one minute), eight (five), and nine (ten).

**Figure 1:**
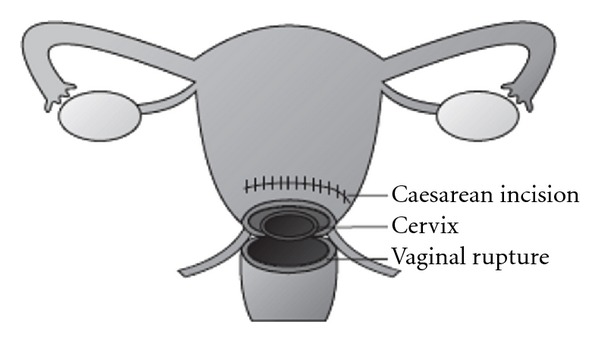
Anterior view of the patient's uterus, showing the vaginal rupture and exposed cervix.

Until this point the LSCS was routine, with no abnormalities noted. The uterus was closed in one layer, with persistent bleeding from an extension of the left uterine incision, near the left ureter. The ureter was thought to have been clamped during the repair, so assistance was sought from urology, who reflected the ureter and bladder off the uterus. This exposed an avulsion tear of the anterior vaginal wall, extending bilaterally to the uterosacral ligaments. The intact, dilated cervix was entirely visible through the tear. (see Figure 1). There was significant bleeding from the vaginal tear as well as from the (intact but friable) posterior bladder wall. Opinion was sought from a second Obstetrician, with consensus that the degree of damage and bleeding warranted a total hysterectomy. This was performed with the assistance of a specialist gynaecological oncologist. A left ureteric stent was also placed by the urology team once the bleeding was controlled.

The patient had a total blood loss of 2500 mL, with her haemoglobin recorded as low as 67 g/L. During the operation she received 6 U of packed red blood cells, 2 U of fresh frozen plasma, 1 L of albumin and 8 L of crystalloid. She also required a metaraminol infusion to maintain her blood pressure above 90/60 mmHg. The total anaesthetic time was seven hours. Postoperatively, the patient was transferred to the intensive care unit, where she remained intubated and ventilated until the next morning. Despite this, she recovered well and was discharged home on day eight.

Histopathology of the patients' uterus revealed that the previous LSCS scar was close to the site of the most recent LSCS incision, that is, 5 cm superior to the site of the rupture.

## 3. Discussion

Worldwide, vaginal trauma sustained during the first stage of labour is often seen in patients with no or poor intrapartum care and manifests as obstetric fistulae secondary to obstructed labour. However, this is exceedingly rare in developed world [2]. Even rarer is the occurrence of vaginal rupture. Review of the literature (Medline, 19/12/2011) revealed only two cases of primary vaginal rupture during labour.

 In one case, a patient with known vaginal atresia experienced a large tear in her posterior vaginal wall, whilst in spontaneous labour at 28 weeks of gestation [3]. In the other case, a patient was induced with oxytocin at 39 weeks of gestation and suffered a rupture of her anterior vaginal wall and posterior bladder wall, during a prolonged second stage [4]. In both cases, the rupture was only discovered at caesarean section, after delivery of the baby.

These two cases of vaginal rupture were attributed to vaginal atresia and prolonged second stage (with oxytocin use), respectively. However, in the case presented above, no cause was identified. Additionally, with the exception of her LSCS scar (which was not involved in the rupture), the patient had none of the risk factors associated with uterine rupture, such as induction or augmentation of labour, obstructed labour, grand multiparity, or placenta percreta [1]. The temporal proximity of the amniotomy and vaginal rupture may suggest causation; however, review of the literature (Medline, November 24, 2011) did not identify any other documented cases of vaginal or uterine rupture immediately following amniotomy. Additionally, a 2006 literature review stated that, even in patients with a uterine scar, “there are no reports of risks associated with membrane stripping or amniotomy” [5].

In the three cases of vaginal rupture described, none was diagnosed prior to laparotomy. However, a common sign between them is the presence of decelerations on the cardiotocograph. This is also the most common indicator of uterine rupture [6].

## 4. Conclusion

Whilst cases such as the one described above are exceptionally rare, it is an important reminder to obstetricians that concealed ruptures of both the vagina and uterus do occur and must be considered in clinical situations where another explanation is not apparent.
